# The Path Forward in MF: Small Molecules in the Limelight

**DOI:** 10.3390/cancers18091370

**Published:** 2026-04-25

**Authors:** Elisabetta Abruzzese, Malgorzata Monika Trawinska, Simona Bernardi, Alessandra Checcoli, Martina Canichella

**Affiliations:** 1 Hematology, St. Eugenio Hospital, Tor Vergata University, ASL Roma2, 00144 Rome, Italymartina.canichella@aslroma2.it (M.C.); 2 Bone Marrow Transplant Unit, ASST Spedali Civili, Department of Clinical and Experimental Sciences, University of Brescia, 25123 Brescia, Italy; simona.bernardi@unibs.it; 3 Pharmacy, St. Eugenio Hospital, ASL Roma2, 00144 Rome, Italy; alessandra.checcoli@aslroma2.it

**Keywords:** myelofibrosis, ruxolitinib, JAK inhibitor resistance, imetelstat, pelabresib, navtemadlin, luspatercept, parsaclisib, nuvisertib, disease-modifying therapy, small molecules

## Abstract

Myelofibrosis (MF) is a chronic myeloproliferative neoplasm characterized by progressive bone marrow fibrosis, extramedullary hematopoiesis (particularly symptomatic splenomegaly), constitutional symptoms, progressive cytopenias, and, in a subset of patients, leukemic transformation. The advent of the JAK1/2 inhibitor ruxolitinib has revolutionized the management of MF, substantially improving splenomegaly, symptom burden, and, in some settings, overall survival. However, a substantial percentage of patients fail to achieve sustained benefit, are intolerant, or become refractory; real-world and clinical trial data indicate that approximately half of treated patients discontinue ruxolitinib treatment within 3 years and up to approximately 75% within 5 years, with poor outcomes after discontinuation (median survival in several series is approximately 12–14 months). In recent years, several new small molecules that act beyond the JAK-STAT axis have emerged in clinical development. These include agents targeting telomerase (imetelstat), epigenetic regulation via BET inhibition (pelabresib/CPI-0610), the MDM2-p53 axis (navtemadlin/KRT-232), erythroid maturation and the bone marrow microenvironment (luspatercept), PI3K signaling (parsaclisib), and PIM inhibitors (nuvisertib). Early clinical data show promising results for symptom and splenic control in specific settings and, importantly, suggest potential disease-modifying activity (improvements in marrow fibrosis and molecular responses) for some compounds. This review summarizes the biological rationale, key clinical data (efficacy and safety), ongoing randomized trials, and remaining knowledge gaps for these non-JAK small molecules in MF and offers practical considerations for integrating them into contemporary treatment algorithms.

## Simple Summary

Myelofibrosis (MF) is a chronic blood cancer characterized by bone marrow fibrosis, anemia, splenomegaly, and systemic symptoms. While JAK inhibitors have significantly improved symptom control, many patients eventually develop resistance or intolerance, and these agents have limited impact on disease progression. In recent years, several novel small molecules targeting pathways beyond JAK–STAT signaling have emerged. These include agents acting on telomerase, epigenetic regulation, apoptotic pathways, inflammatory signaling, and erythropoiesis. This review provides an updated overview of ongoing and recently reported clinical trials, highlighting the evolving therapeutic landscape and the potential of these agents to move beyond symptom control toward more disease-directed strategies, while addressing their long-term efficacy and impact on survival.

## 1. Introduction

Myelofibrosis (MF) is a clonal chronic myeloproliferative neoplasm characterized by bone-marrow fibrosis, extramedullary hematopoiesis with prominent splenomegaly, constitutional symptoms (fatigue, weight loss, night sweats, fever), progressive cytopenias (notably anemia and thrombocytopenia), thrombotic and hemorrhagic complications, and an increased risk of leukemic transformation [1,2,3]. MF exhibits a heterogeneous clinical course ranging from indolent to rapidly progressive disease; validated prognostic models such as International prognostic scoring system (IPSS) and Dynamic International Prognostic Scoring System (DIPSS) -and their derivatives- are used to estimate survival and to guide therapeutic decisions, including selection of candidates for allogeneic hematopoietic stem cell transplantation (allo-HSCT), the only potentially curative option [4,5,6,7,8].

The approval of the JAK1/JAK2 inhibitor ruxolitinib represented a major therapeutic advance in MF. Randomized trials and subsequent real-world experiences demonstrated substantial and durable reductions in spleen volume, significant improvement in MF-related symptom burden and quality of life, and a survival advantage in some trial cohorts [9]. Nevertheless, important limitations remain: a substantial proportion of patients fail to obtain durable clinical benefit, treatment- or disease-related cytopenias may limit drug exposure or necessitate discontinuation, and anemia, when present, typically worsens during ruxolitinib therapy, resulting in an increased number of transfusion-dependent patients as well as higher transfusion frequency. In addition, real-world and trial datasets report high rates of ruxolitinib discontinuation—roughly 50% within three years and up to ~75% by five years in some series—with poor outcomes after discontinuation (median overall survival frequently in the low-teens of months in retrospective cohorts) [10,11]. Moreover, clonal evolution and acquisition of high-risk molecular lesions contribute to disease progression despite JAK-STAT blockade [12,13,14,15].

These limitations define unmet needs in MF treatment: (i) therapies capable of extending survival and inducing disease modification (reduction of marrow fibrosis and/or clonal burden); (ii) alternative options for patients refractory to or relapsing after JAK inhibitor therapy; and (iii) approaches acting through non-JAK mechanisms that can be combined with JAK inhibitors or other targeted agents [16]. In this setting, several classes of small molecules targeting pathways outside the JAK–STAT axis have been applied in clinical trials. Among these, imetelstat, a telomerase inhibitor with phase II evidence of potential disease-modifying activity and an ongoing phase III program (IMpactMF) in higher-risk, JAK-inhibitor-refractory MF [17,18,19]; pelabresib, a bromodomain and extraterminal domain (BET) inhibitor evaluated as monotherapy and in combination with ruxolitinib in the MANIFEST program with encouraging clinical and biomarker results [20,21,22]; navtemadlin, an oral MDM2 inhibitor that reactivates p53 signaling in TP53-wildtype MF and has shown activity in relapsed/refractory cohorts [23]; luspatercept, a TGF-β superfamily ligand trap that enhances late erythroid maturation and has demonstrated benefit for MF-associated anemia [24], particularly in combination with JAK inhibitors [25,26]; parsaclisib, a selective PI3Kδ inhibitor developed as an add-on to ruxolitinib in patients with suboptimal response, with phase II/III evaluations showing spleen and symptom benefits in selected dosing schemas [27,28,29]. Finally, nuvisertib, an oral PIM1 kinase inhibitor that targets JAK-independent inflammatory and survival signaling and has demonstrated clinically meaningful symptom improvement with associated cytokine modulation in phase I/II studies in patients with myelofibrosis [30].

The main characteristics of the mentioned molecules are reported in Table 1.

This review aims to summarize the mechanistic rationale for each agent class and to report the clinical evidence indeed we discuss ongoing randomized studies and practical considerations for sequencing and combination strategies. We conclude by outlining the outstanding clinical and biological questions and the future directions that will guide the incorporation of non-JAK small molecules into contemporary MF treatment strategies.

## 2. Luspatercept

Anemia is one of the most clinically predominant symptoms of MF contributing to fatigue, reduction of exercise tolerance and quality of life, and increased transfusion dependence and its complications; indeed, the fatigue could often limit the use and dosing of JAK inhibitors such as ruxolitinib. Luspatercept (ACE-536) is an erythroid maturation agent that acts as a ligand trap for select TGF-β superfamily members, reducing SMAD2/3 signaling and thereby enhancing late-stage erythroid maturation. This mechanism -distinct from erythropoiesis-stimulating agents and supportive transfusions- provides a biologically plausible approach to improve hemoglobin and reduce transfusion needs in MF patients with ineffective erythropoiesis [31,32,33].

The clinical development of luspatercept in MF has been led by the open-label phase II ACE-536-MF-001 study (NCT03194542), which enrolled patients across four cohorts defined by transfusion dependence and concurrent JAK inhibitor use. The trial enrolled both transfusion-dependent and transfusion-independent patients, as well as individuals receiving or not receiving ruxolitinib [26]. The ACE-536-MF-001 program produced two clinically relevant observations. First, luspatercept induced hemoglobin increases and reductions in transfusion burden across cohorts; the greatest absolute benefits were seen in transfusion-dependent patients who were receiving concomitant ruxolitinib, where a consistent proportion of patients achieved transfusion independence for predefined intervals and many more experienced ≥50% reductions in transfusion requirements [34,35]. Second, luspatercept did not appear to worsen splenomegaly or enhance extramedullary erythropoiesis, and was generally tolerable: the safety profile in MF mirrored that seen in other indications (MDS with ring sideroblasts, β-thalassemia), with common adverse events including hypertension, bone/musculoskeletal pain, and dizziness, and without a signal for excess marrow fibrosis or overt pro-thrombotic toxicity in the reported cohorts. Exploratory biomarker analyses from the trial sought predictors of response (for example, baseline erythropoietic indices and iron-related markers), but robust, prospectively validated biomarkers of luspatercept benefit in MF remain to be established [34].

On the basis of these encouraging phase II results, the double-blind, randomized, placebo-controlled phase III trial INDEPENDENCE (NCT04717414) was initiated to test whether luspatercept could lead to transfusion independence in MF patients who remain transfusion-dependent while on stable JAK-inhibitor therapy. The trial’s primary endpoint—proportion of patients achieving ≥12 consecutive weeks of transfusion independence within the first 24 weeks—reflected an outcome that is patient-centered, clinically relevant, and regulatory-aligned. Secondary endpoints included reduction in transfusion burden, durability of response, changes in hemoglobin, symptom scores, spleen response and safety [36,37].

The phase III INDIPENDENCE trial demonstrated that, while luspatercept produced clinical improvements in several secondary endpoints (for example reductions in transfusion burden and increases in mean hemoglobin), it missed the primary endpoint regarding the proportion achieving the 12-week transfusion independence window in the entire primary analysis population [38]. These results could be related to the heterogeneity of the subgroups and the patients who showed more favorable hematologic improvements—a finding consistent with the phase II observations- were those with concomitant ruxolitinib. Anywhere the results of luspatercept in reduction of transfusion burden and improvement of hemoglobin level may still be clinically valuable even if the stringent primary endpoint was not met [38]. Longer follow-up and full peer-reviewed data releases (including prespecified subgroup and sensitivity analyses, duration of benefit, and safety details) are awaited to clarify the magnitude and durability of benefit and to identify populations most likely to derive clinically meaningful improvement.

Despite the heterogeneous results from INDEPENDENCE, the overall body of evidence indicates that luspatercept is capable of producing clinically relevant improvements in anemia for a subset of patients, an important unmet need in MF. However, it is also clear that anemia correction in MF requires multifaceted approaches targeting both disease biology and treatment-related mechanisms. In this respect, momelotinib, with its dual JAK1/JAK2 and ACVR1 inhibition leading to suppressed hepcidin and improved iron availability, represents a complementary strategy that inherently addresses MF-associated anemia and has demonstrated anemia-related benefits in multiple randomized trials [39,40]. Its approval has further expanded the therapeutic armamentarium for MF-related anemia and underscores the need for integrated, mechanism-informed approaches to overcome the erythropoietic dysfunction characteristic of this disease.

## 3. Parsaclisib

Parsaclisib (INCB050465), a potent and selective PI3Kδ inhibitor, has been explored as combination therapy in patients with MF with suboptimal responses to ruxolitinib. Activation of the PI3K–AKT pathway has been implicated as a compensatory survival mechanism in MF cells exposed to chronic JAK inhibition, providing a biological rationale for dual pathway suppression. In a multicenter phase II study, parsaclisib added to stable ruxolitinib therapy demonstrated clinically meaningful activity, with spleen volume reductions and symptom improvements observed across different dosing schedules, although grade 3–4 thrombocytopenia emerged as a notable treatment-related toxicity [28,41]. These encouraging data led to the initiation of two phase III trials within the LIMBER program, designed to evaluate parsaclisib in patients with persistent splenomegaly and symptomatic burden despite JAK inhibition. However, the LIMBER-304 trial was discontinued after an interim analysis indicated a very low probability of achieving the primary endpoint, despite the absence of new safety concerns [42]. Subsequently, the first-line LIMBER-313 trial was also closed [41]. Collectively, these findings suggest that while PI3Kδ blockade can augment ruxolitinib activity in selected subgroups, the strategy has not translated into robust clinical benefit. The experience with parsaclisib highlights the biological complexity of MF and underscores the need for more integrated, biomarker-driven, or multi-pathway approaches to overcome resistance and heterogeneous responses observed with JAK inhibitor therapy [43].

## 4. Pelabresib

Pelabresib (CPI-0610) is an oral bromodomain and extraterminal (BET) protein inhibitor that modulates epigenetic transcriptional programs central to myelofibrosis (MF) pathobiology, including aberrant megakaryopoiesis, pro-inflammatory cytokine signaling, and fibrogenic remodeling of the bone marrow microenvironment. BET proteins act as chromatin readers regulating NF-κB-driven inflammatory transcription and oncogenic gene expression; their inhibition has been shown in preclinical MF models to reduce cytokine production, splenomegaly, and marrow fibrosis, with synergistic effects when combined with JAK–STAT inhibition, providing a strong mechanistic rationale for combination therapy with ruxolitinib [44,45].

Clinical activity of pelabresib was first demonstrated in the phase II MANIFEST study (NCT02158858), in which pelabresib plus ruxolitinib produced high rates of spleen and symptom responses in both JAK inhibitor–naïve patients and those with suboptimal responses to ruxolitinib. In the frontline cohort, pelabresib plus ruxolitinib achieved SVR35 in 68% and TSS50 in 56% at Week 24. Cytopenias, primarily anemia, and thrombocytopenia were the most common adverse events but were generally manageable [21].

These results supported the phase III MANIFEST-2 trial (NCT04603495), which compared pelabresib plus ruxolitinib with placebo plus ruxolitinib in JAK inhibitor–naïve MF, confirming the clinical relevance of this approach.

In fact, MANIFEST-2 met its primary endpoint, with a significantly higher SVR35 rate in the pelabresib arm (65.9%) versus control (35.2%), representing one of the strongest spleen responses reported in frontline MF therapy [35,36]. Symptom responses, bone-marrow fibrosis improvement, and modulation of inflammatory biomarkers were also encouraging, and safety findings remained consistent with earlier studies [20].

At ASH 2025, updated 96-week MANIFEST-2 data showed that the clinical benefits of pelabresib were deep and durable, with sustained spleen volume reductions, persistent symptom improvement, and a higher proportion of patients achieving combined SVR35 and TSS50 responses compared with ruxolitinib monotherapy. In fact, SVR35 response was observed in 91.5% of patients receiving pelabresib plus ruxolitinib versus 57.5% with ruxolitinib alone, corresponding to 45.3% versus 30.1% of the intent-to-treat population, respectively, and confirming sustained spleen responses over time. Symptom improvement was also durable, with a mean absolute change in total symptom score (TSS) of −15.07 versus −12.48 at Week 96 and TSS50 achieved by 36.9% versus 28.2%. Notably, dual SVR35 and TSS50 responses, a composite endpoint associated with meaningful clinical benefit, were reported in 31.8% of patients treated with pelabresib plus ruxolitinib compared with 15.7% receiving ruxolitinib alone.

Importantly, long-term follow-up demonstrated consistent improvements in bone marrow morphology, including higher rates of ≥1-grade fibrosis regression, reduced megakaryocyte density, and increased erythroid progenitor populations, supporting a potential disease-modifying effect. Molecular analyses showed greater reductions in driver mutation variant allele frequency, while progression-free and overall survival curves remained favorable with longer follow-up. There were no emerging long-term safety signals, and the initially reported imbalance in leukemic progression in the pelabresib arm decreased over time, with observed rates aligning with those expected for myelofibrosis. These results represent the longest follow-up to date of a randomized combination trial in myelofibrosis and collectively reinforce pelabresib plus ruxolitinib as a first-line, mechanism-based combination strategy with the potential to alter the disease course in MF [46,47].

## 5. Navtemadlin

Navtemadlin (KRT-232) is an oral antagonist of the MDM2–p53 interaction designed to restore p53-mediated apoptosis in malignant hematopoietic clones retaining wild-type TP53. Given the frequent dysregulation of apoptotic pathways and the persistence of clonal hematopoiesis in MF, pharmacologic reactivation of p53 offers a compelling strategy to induce clonal reduction and potentially disease-modifying effects [23,48].

In the phase II study KRT-232-113, navtemadlin was administered to patients with R/R MF -including those previously treated with JAK inhibitors or ineligible for them- to assess safety, tolerability, and early signals of hematologic and disease-modifying activity [23,49]. The regimen demonstrated a manageable safety profile; notable adverse events included gastrointestinal symptoms and reversible cytopenias, but no unexpected toxicity was reported. Hematologic responses were characterized by reductions in circulating CD34^+^ progenitor counts and decreases in variant allele frequencies (VAF) in a subset of treated patients, supporting the possibility of clonal suppression. Some patients also experienced reduction in splenomegaly and constitutional symptoms, though the magnitude and durability of responses varied among individuals [49]. Collectively, these findings provide proof-of-concept that MDM2 inhibition via navtemadlin can target the malignant clone in MF, potentially offering a therapeutic option beyond JAK-STAT inhibition.

Nevertheless, the data remain preliminary: long-term follow-up, larger cohort validation, and controlled trials are needed to confirm whether the clonal reductions translate into sustained clinical benefit, improved marrow fibrosis, or survival advantage. The KRT-232-113 results highlight both promise and challenges of pro-apoptotic, clone-directed therapy in MF, especially in patients with limited options post-JAK inhibitor failure or ineligibility for standard therapies.

## 6. Nuvisertib

Nuvisertib (TP-3654) is an oral, selective PIM1 kinase inhibitor. PIM1/2/3 (Proviral Integration site for Moloney murine leukemia virus) are constitutively active serine/threonine kinases and key downstream effectors of cytokine and growth factor signaling, particularly the JAK–STAT pathway. Unlike many kinases, PIM proteins do not require activating mutations or phosphorylation; their activity is primarily regulated at the transcriptional and translational levels, resulting in sustained signaling once induced. In myelofibrosis (MF), PIM kinases promote cytokine-driven inflammation, clonal survival, metabolic adaptation, and resistance to JAK inhibition. Preclinical studies show that selective PIM1 knockout prevents MF progression without affecting platelet counts, whereas pan-PIM deletion induces thrombocytopenia, supporting selective PIM1 inhibition as a rational and potentially safer therapeutic strategy.

In MF, PIM kinases are overexpressed as a consequence of chronic JAK–STAT activation. They are implicated in disease pathogenesis by promoting malignant cell survival, proliferation, and resistance to apoptosis through regulation of targets involved in cell cycle progression, MYC stabilization, mTOR signaling, and anti-apoptotic pathways (e.g., BAD phosphorylation, Bcl-2-associated agonist of cell death). In addition, PIM kinases contribute to the inflammatory milieu characteristic of MF by enhancing cytokine production and sustaining JAK-independent signaling loops.

Importantly, PIM kinase activity persists despite pharmacologic JAK inhibition, providing a functional explanation for incomplete or transient responses to JAK inhibitors and for the persistence of symptoms and disease activity in many patients. This JAK-independent role makes PIM kinases particularly attractive therapeutic targets in MF, as their inhibition has the potential to suppress residual inflammatory signaling, overcome adaptive resistance mechanisms, and complement the clinical effects of JAK inhibitors [50,51].

By inhibiting PIM-1 kinase, nuvisertib aims to suppress JAK-independent signaling, reduce inflammatory cytokine production, and restore apoptotic sensitivity in malignant hematopoietic cells [52].

At ASH 2024, updated in 2025, results from the phase 1/2 study of nuvisertib monotherapy, and the preliminary data of the combination with momelotinib in patients with intermediate- or high-risk MF were presented. In the evaluable cohort treated in monotherapy at the recommended dose of 720 mg twice daily, clinically meaningful symptom improvement was observed, with 45% of patients achieving a ≥50% reduction in total symptom score (TSS50) at any time on treatment (9/20 patients).

Symptom responses were accompanied by biological activity, including modulation of circulating inflammatory cytokines, supporting the proposed mechanism of action. Exploratory analyses suggested an association between cytokine changes and one-year overall survival, indicating that PIM kinase inhibition may impact not only symptom burden but also disease biology. This was supported by the observation that 38% of evaluable patients (13/34) showed >=1 grade improvement in marrow fibrosis. MIP-1b and TNF-RI, respectively a pro-inflammatory cytokine and the primary receptor mediating TNFa-induced pro-inflammatory signaling, were also identified as candidate biomarkers of bone marrow fibrosis improvement. Treatment was generally manageable, with no unexpected safety signals reported in this heavily pretreated population. Anemia was manageable since Hb remained stable during the treatment, and 25% of patients showed an increase >1 g/dL Hb [30].

Collectively, these data support further clinical development of nuvisertib providing a strong rationale for its evaluation in combination strategies, particularly alongside JAK inhibitors, to address persistent inflammation and non-JAK-dependent disease drivers in MF.

Preliminary data from the phase 1/2 study of nuvisertib, escalated dose, in combination with momelotinib (200 mg QD) to improve response rates with primary endpoint of safety and tolerability were presented for the first 18 patients enrolled at ASH 2025. The combination showed both symptoms and spleen responses obtained early during treatment and Hb improvement in 56% (9/16) evaluable patients.

## 7. Imetelstat

Imetelstat (GRN163L) is a first-in-class telomerase inhibitor that targets the RNA template (hTR) of telomerase, thereby competitively inhibiting telomere elongation in malignant hematopoietic progenitor cells. Given that telomerase activity is upregulated in myelofibrosis (MF) and contributes to clonal proliferation and disease persistence, its inhibition represents a compelling disease-modifying strategy aimed at reducing malignant clone fitness rather than solely controlling downstream signaling pathways. In addition to its primary mechanism, preclinical data suggest that imetelstat may also modulate JAK–STAT signaling, particularly in CALR-mutant cells, through reduction of JAK2 phosphorylation and inhibition of downstream effectors such as STAT3 and STAT5 [53].

Clinical development of imetelstat in MF has primarily focused on patients with intermediate-2 or high-risk disease who are refractory to or have relapsed after JAK inhibitor therapy. In the phase II IMbark study, imetelstat demonstrated meaningful clinical activity, including symptom improvement, spleen responses, and, notably, evidence suggestive of disease modification. A subset of patients achieved reductions in bone marrow fibrosis grade and decreases in driver mutation variant allele frequency, indicating potential effects on the underlying malignant clone. Importantly, median overall survival observed in the higher-dose cohort appeared longer than historically expected in this setting, raising the hypothesis that telomerase inhibition may translate into survival benefit in selected patients [54].

The safety profile of imetelstat was characterized mainly by reversible cytopenias, particularly thrombocytopenia and neutropenia, consistent with its mechanism of action on proliferating hematopoietic cells. Liver function abnormalities and gastrointestinal events were also reported but were generally manageable with dose modifications and supportive care.

These encouraging findings have led to the ongoing phase III IMpactMF trial (NCT04576156), which is evaluating imetelstat versus best available therapy in patients with relapsed/refractory MF after JAK inhibitor treatment. The primary endpoint of overall survival reflects the increasing focus on disease modification rather than symptomatic control alone. If positive, this study may establish imetelstat as the first agent in MF to demonstrate a survival advantage in the post-JAK inhibitor setting and could redefine treatment goals toward clonal and biological disease control [18].

Overall, imetelstat represents a unique therapeutic approach in MF, targeting a fundamental mechanism of cellular immortality. Its potential to impact survival and disease biology distinguishes it from other non-JAK small molecules and supports its integration into future mechanism-driven treatment strategies, particularly in patients with limited options after JAK inhibitor failure.

**Figure 1:**
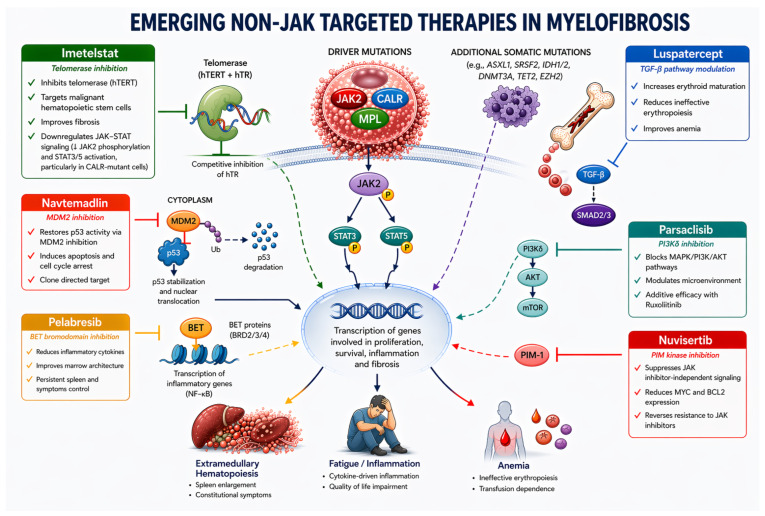
Emerging non-JAK-targeted therapies in myelofibrosis.

The commented therapeutic molecules, their mechanisms of action, and effects are summarized in Figure 1.

Myelofibrosis is driven by constitutive activation of JAK–STAT signaling resulting from canonical driver mutations in JAK2, CALR, or MPL, leading to phosphorylation of JAK2 and activation of STAT3/STAT5, while additional somatic mutations (e.g., ASXL1, SRSF2, IDH1/2) contribute to disease progression and adverse prognosis. Beyond JAK inhibition, several investigational agents target complementary pathogenic pathways at different levels of the signaling network. Imetelstat inhibits telomerase activity and may also modulate JAK–STAT signaling. Pelabresib targets BET bromodomain proteins, regulating NF-κB-dependent transcription. Navtemadlin inhibits the MDM2–p53 interaction, restoring p53-mediated transcriptional activity. Parsaclisib blocks the PI3K–AKT pathway, while nuvisertib inhibits PIM-kinase-mediated survival signaling. Luspatercept modulates erythroid maturation through the TGF-β/SMAD pathway within the bone marrow microenvironment.

By acting on distinct biological mechanisms, these agents aim to address key clinical manifestations of myelofibrosis, including extramedullary hematopoiesis, systemic inflammation and fatigue, and anemia, and may provide disease-modifying potential.

## 8. Discussion

The therapeutic landscape of MF has expanded considerably beyond JAK inhibitors, with several novel small molecules emerging to potentially address unmet clinical needs [55]. These agents address diverse pathogenic mechanisms -ineffective erythropoiesis, survival signaling, epigenetic dysregulation, defective apoptotic control, and compensatory inflammatory pathway- reflecting the multifactorial nature of MF and the heterogeneity of clinical manifestations.

Luspatercept, a ligand trap that enhances late-stage erythroid maturation, has demonstrated significant improvements in transfusion dependence and hemoglobin levels, particularly in patients receiving continuative ruxolitinib treatment [25,26,31,33]. Although the INDEPENDENCE trial did not meet its primary endpoint [37], phase II data and real-world reports support the biologic validity of targeting ineffective erythropoiesis. These findings underscore the principle that anemia in MF is mechanistically distinct from splenomegaly or symptom burden and may require dedicated therapies. Importantly, strategies like momelotinib, which modulate ACVR1-mediated hepcidin pathways, complement these approaches by simultaneously targeting disease-related anemia and systemic inflammation [39].

Parsaclisib, a selective PI3Kδ inhibitor, represents an attempt to overcome compensatory survival signaling in patients with suboptimal responses to ruxolitinib [28]. Phase II studies suggested that spleen and symptom responses can be enhanced in selected patients [56]; however, the early discontinuation of phase III LIMBER-304 and LIMBER-313 trials illustrates the challenges of translating pathway-based rationale into robust clinical benefit at a population level. These outcomes highlight the complexity of MF biology, including clonal heterogeneity, cytokine-driven microenvironmental support [57], and patient-specific factors such as baseline cytopenias, which can limit tolerability and efficacy.

Pelabresib, a BET inhibitor, offers a compelling complementary mechanism by targeting epigenetic drivers of fibrosis and inflammation [22,44,45,47]. The MANIFEST-1 and MANIFEST-2 trials demonstrated that combination therapy with ruxolitinib yields deeper spleen responses, symptom improvements, and potential signals of disease modification, including bone-marrow fibrosis regression and allele burden reduction [20,21,45,46]. The Week 96 long-term data from MANIFEST-2, recently presented by Rampal and colleagues, have been pivotal in defining the durability of these responses and their clinical relevance in frontline therapy, suggesting that epigenetic modulation may enhance the efficacy of JAK inhibitors beyond symptom control [22].

Navtemadlin, an MDM2 inhibitor, addresses a complementary aspect of MF pathobiology by reactivating p53-mediated apoptosis in TP53-wildtype clones [23]. Early-phase data show clonal suppression, reductions in circulating progenitors, and symptomatic improvement in relapsed/refractory patients, illustrating the potential of pro-apoptotic, clone-directed therapy, particularly in heavily pretreated patients [23,49]. While preliminary, these findings highlight the promise of targeting core survival pathways that are independent of JAK-STAT signaling and may be particularly relevant in heavily pretreated populations.

The clinical and biological activity observed with nuvisertib supports the therapeutic relevance of targeting PIM kinases in MF [58]. Persistent PIM kinase signaling despite JAK inhibition represents a key mechanism of residual inflammation and disease maintenance, and its disruption may help address important unmet needs in patients with suboptimal or waning responses to JAK inhibitors. The observed improvements in symptom burden, modulation of inflammatory cytokines, and signals of bone marrow fibrosis regression suggest that PIM kinase inhibition may extend beyond symptomatic benefit and impact underlying disease biology. The identification of MIP-1β and TNF-RI as candidate biomarkers of fibrosis improvement further supports a mechanistic link between inflammatory signaling and marrow remodeling in MF. Notably, the impact on hemoglobin levels, both in monotherapy and in combination with momelotinib, is particularly relevant in a disease where anemia remains a major clinical challenge [59]. These findings suggest a potential complementary role for nuvisertib alongside JAK inhibitors, particularly those with anemia-sparing properties.

Imetelstat further expands this therapeutic armamentarium by targeting telomerase, a key mechanism sustaining clonal proliferation and replicative immortality in MF. Unlike agents primarily modulating downstream signaling or the inflammatory microenvironment, early data suggest a potential disease-modifying effect. However, these findings remain preliminary and require confirmation.

In addition to pathway-directed therapies, strategies aimed at directly targeting disease-driving mutations are under active investigation. Selective and type II JAK2 inhibitors, as well as emerging allosteric inhibitors and JAK2 degraders, are designed to improve specificity compared with first-generation ATP-competitive inhibitors [60,61]. Novel strategies targeting CALR-mutant disease, including mutant CALR-directed antibodies, are also being explored, further supporting a shift toward mutation-specific therapeutic approaches [62]. While these approaches remain in early clinical development and clear superiority over approved JAK inhibitors has yet to be demonstrated, their integration with non-JAK-directed small molecules holds promise to reshape future therapeutic strategies and improve outcomes for patients with MF.

## 9. Conclusions

In conclusion, JAK inhibition remains the foundational therapy for symptomatic MF, providing robust control of splenomegaly and symptom burden. However, limitations such as incomplete efficacy, treatment-emergent cytopenias, and minimal impact on disease progression, highlight the pressing need for complementary therapeutic strategies.

The emerging small molecules discussed herein—luspatercept, parsaclisib, pelabresib, navtemadlin, nuvisertib and imetelstat—collectively exemplify the potential of targeting non-JAK pathways to address these unmet needs. By enhancing late-stage erythropoiesis, modulating survival and inflammatory pathways, altering epigenetic programs, and restoring p53-mediated apoptosis, these agents offer not only symptomatic benefit but also the possibility of true disease modification, including improvements in marrow fibrosis, clonal burden, and inflammatory milieu, while nuvisertib adds a peculiar dimension by addressing persistent JAK-independent signaling and inflammation.

The development of these novel agents highlights the evolving complexity and precision of MF therapy. Integrating molecular and clinical biomarkers will be critical to identify patients most likely to benefit, optimize sequencing, and guide rational combination strategies while minimizing toxicity. As long-term results mature, a more comprehensive understanding of response durability, survival impact, and disease modification potential is anticipated. Collectively, these advances may shift MF therapy from primarily symptom-directed management toward a mechanism-driven, multi-targeted approach addressing both clinical manifestations and underlying disease biology.

## Figures and Tables

**Table 1:** Reports the main drugs developed in the setting of MF in recent years, their targets, and the most important observations obtained from the related clinical trials.

Drug	Code Name	Target	MF Clinical Setting	Key Efficacy Signals	Comparative Positioning vs. JAK Inhibitors ^9^	Unmet Need Addressed
Pelabresib	CPI-0610	BET proteins	Phase 3; frontline MF + ruxolitinib ^3^	Higher SVR35 ^1^ and TSS50 ^2^; fibrosis and anemia signals ^3^	Frontline combination to deepen/prolong JAK inhibitor benefit ^3^	Suboptimal depth/durability of response with JAK inhibition alone
Navtemadlin	KRT-232	MDM2	Phase 2–3; post-JAK inhibitor MF ^4^	Spleen and symptom responses in TP53–WT patients ^4^	Non-JAK option after JAK inhibitor failure ^4^	Limited options after JAK inhibitor discontinuation
Parsaclisib	INCB050465	PI3Kδ	Phase 2; add-on to ruxolitinib ^5^	Incremental symptom and modest spleen responses ^5^	Adjunctive therapy to enhance JAK inhibitor symptom control ^5^	Persistent symptom burden despite JAK inhibition
Imetelstat	GRN163L	Telomerase (hTERC)	Phase 3; relapsed/refractory MF ^6^	Durable responses in subset; survival and molecular signals ^6^	Potential disease-modifying agent post-JAK inhibitor ^6^	Lack of therapies altering disease biology and survival
Luspatercept	ACE-536	TGF-β ligand trap	Phase 2–3; MF-associated anemia ^7^	Increased hemoglobin; higher transfusion independence ^7^	Supportive agent complementing JAK inhibitors ^7^	JAK-inhibitor-related and disease-related anemia
Nuvisertib	TP-3654	PIM1 kinase	Phase 2; MF + ruxolitinib ^8^	Spleen and symptom improvements in combination ^8^	Add-on strategy to augment or restore JAK inhibitor efficacy ^8^	Incomplete responses and emerging JAK inhibitor resistance

Footnotes: ^1^ SVR35 = ≥35% spleen volume reduction by MRI/CT. ^2^ TSS50 = ≥50% reduction in total symptom score (MPN-SAF or equivalent). ^3^ Pelabresib + ruxolitinib: MANIFEST-2 (Phase 3; NCT04603495); MANIFEST Phase 2 for add-on/combo cohorts. ^4^ Navtemadlin (KRT-232): BOREAS (NCT0679135) Phase 3, relapsed/refractory MF. ^5^ Parsaclisib (INCB050465): Phase 2 add-on study in ruxolitinib suboptimal responders. ^6^ Imetelstat (GRN163L): IMpactMF (NCT04576156) Phase 3, post-JAK inhibitor MF. ^7^ Luspatercept (ACE-536): Phase 2–3 studies (e.g., INDEPENDENCE, NCT04717414) in MF-associated anemia. ^8^ Nuvisertib (TP-3654): Phase 2, mainly in combination with ruxolitinib (NCT04176198). ^9^ JAK inhibitor settings reflect relative positioning; JAK inhibitors remain the backbone of MF therapy.

## Data Availability

No new data has been generated for this manuscript.
